# Why Gayborhoods Matter: The Street Empirics of Urban Sexualities

**DOI:** 10.1007/978-3-030-66073-4_4

**Published:** 2020-11-30

**Authors:** Amin Ghaziani

**Affiliations:** 17 Department of Architecture and Design, Alfred State University of New York, New York, USA; 18 Department of Urban and Regional Planning, University at Buffalo, Buffalo, NY USA; Amin Ghaziani University of British Columbia, Vancouver, BC Canada

**Keywords:** Urban sexualities, Technology, Gay neighborhoods, LGBTQ+ safe spaces

## Abstract

Urbanists have developed an extensive set of propositions about why gay neighborhoods form, how they change, shifts in their significance, and their spatial expressions. Existing research in this emerging field of “gayborhood studies” emphasizes macro-structural explanatory variables, including the economy (e.g., land values, urban governance, growth machine politics, affordability, and gentrification), culture (e.g., public opinions, societal acceptance, and assimilation), and technology (e.g., geo-coded mobile apps, online dating services). In this chapter, I use the residential logics of queer people—why they in their own words say that they live in a gay district—to show how gayborhoods acquire their significance on the streets. By shifting the analytic gaze from abstract concepts to interactions and embodied perceptions on the ground—a “street empirics” as I call it—I challenge the claim that gayborhoods as an urban form are outmoded or obsolete. More generally, my findings caution against adopting an exclusively supra-individual approach in urban studies. The reasons that residents provide for why their neighborhoods appeal to them showcase the analytic power of the streets for understanding what places mean and why they matter.

## Introduction: Gayborhood Studies

The association between sexuality and the city is as established experientially as it is affirmed in the academy—from sexological counts of sexual practices to thick ethnographic descriptions of the moral regions of urban sexual worlds (Kinsey et al. 1948; Park 1915; Park and Burgess 1925; Thomas 1907). Although the spatial expressions of queerness are a relatively recent object of inquiry, I see foundational works in anthropology (Newton 1993; Rubin 1998; Weston 1995), Black queer studies (Nero 2005), economics (Black et al. 2002), feminist studies (Rupp 2009; Wolfe 1979), geography (Brown 2014; Hubbard 2012; Nash and Gorman-Murray 2014), history (Aldrich 2004; Chauncey 1994; Heap 2003; Kennedy and Davis 1993), sociology (Castells 1983; Laumann et al. 2004), and urban studies (Delany 1999; Fischer 1975) as part of a distinct field of “ gayborhood studies” (Ghaziani 2014b, 2015b, 2019c). Research in this area focuses on the properties of urban gay districts, including their spatial, historical, prototypical, institutional, and comparative features.^1^ Today, new works are published at too rapid a rate for me to capture in just one citation (e.g., Baldor 2018; Bitterman 2020; Callander et al. 2020; Forstie 2019; Stone 2018).

The field of gayborhood studies consists of four major streams. One area of research focuses on the origins and ontology of these districts (Compton and Baumle 2012). Scholars ask why gayborhoods first formed (Castells and Murphy 1982; Knopp 1997; Lewis 2013), how they have changed over time (Kanai and Kenttamaa-Squires 2015; Rushbrook 2002; Stryker and Van Buskirk 1996), their cultural significance for queer people (Doan and Higgins 2011; Greene 2014; Orne 2017), why they appeal to heterosexuals (Brodyn and Ghaziani 2018; Ghaziani 2019d), and their diverse spatial expressions ( Brown-Saracino 2018; Ghaziani 2019a; Whittemore and Smart 2016). Regardless of whether they ask about origins, change, resonance, inter-group dynamics, or spatial variability, scholars who work in this area generally propose macro-structural arguments. For example, standard scholarly accounts point to economic forces, especially gentrification, to explain why gayborhoods form and change (Christafore and Leguizamon 2018; Collins 2004; Ruting 2008). Culturalists respond by arguing that gayborhoods are “a spatial response to a historically specific form of oppression” (Lauria and Knopp 1985: 152). When the nature of oppression changes, so too should the spatial response (Andersson 2019; Ghaziani 2014b). A small but vibrant area in this first group asks how a post-gay turn (Ghaziani 2011) affects these districts (Forbes and Ueno 2019; Forstie 2018; Ghaziani 2015a; Hartless 2018).

A second research stream investigates the organizational profile of gayborhoods. In earlier studies, scholars argued that the institutional elaboration of queer communities made them quasi-ethnic in character and composition (Epstein 1987; Murray 1979). This prompted follow-up questions about whether gay districts resemble ethnic ghettos (Levine 1979; Wirth 1928) and if gay bars are better conceptualized as private (Weightman 1980) or closet-like spaces (Brown 2000). From here, researchers documented the growth of public LGBTQ organizations (Armstrong 2002), pride parades (Bruce 2016), and the globalization of queer spaces (Martel 2018). Similar to the first stream, those who work in the second also favor analytic approaches that are abstracted from the streets, including debates about shifting political logics, theories of field formation, and the interplay between global templates and local variations of urban sexualities .

A third stream focuses on the effects of technology. Geo-coded mobile apps enable same-sex sexual partner selection to occur with greater ease outside the context of any one neighborhood. Location-based digital apps facilitate sexual transactions, and users can construct networks of intimacy across the city (Race 2015) according to their tastes (Clay 2018) and personal preferences—but researchers find that these so-called “preferences” are also coded forms of sexual racism (Callander et al. 2016; Han and Choi 2018; Robinson 2015). A common argument is that geo-aware applications like Grindr decenter placemaking efforts (Collins and Drinkwater 2017; Roth 2016). One study of seventeen cities found that in every single one, “the virtual gay community was larger than the offline physical community” (Rosser et al. 2008: 588). Other researchers use the spatial concentration of men who have sex with other men, and their online activities, to track the spread of HIV and other sexually transmitted infections (Card et al. 2018; Salway et al. 2019). These findings have triggered debates about the uneven effects of technology (Blackwell et al. 2015). Some researchers show that people use technology creatively to imagine new spaces away from the gayborhood (Wu and Ward 2017), while others argue that apps reproduce inequalities (Conner 2018).

Rather than origins, organizations, and technology, researchers who work in a fourth stream of gayborhood studies document demographic changes (Morales 2018; Spring 2013) and consider their effects on community-building and placemaking efforts ( Brown-Saracino 2011; Casey 2004; Ghaziani and Stillwagon 2018; Renninger 2019). A topic of particular concern is the fate of gay bars. In San Francisco, Mattson (2015) shows that the popularity of gay bars among straight people has nearly wiped them out; their numbers dropped from thirteen to three in just eleven years. The decline in San Francisco is part of an international pattern. From 2006 to 2016, the number of LGBTQ bars, pubs, and nightclubs in London, UK plummeted by 58%, falling from 125 venues to fifty-three (Campkin and Marshall 2017). This prompted the mayor to appoint a “night czar” to oversee the capital’s £26.3 billion nighttime economy (Ghaziani 2019b). In the United States, the number of gay bar listings in the *Damron Guide* fell by 36.6% (Mattson 2019). Researchers have documented similar “structural declines” in France, Denmark, Sweden, Amsterdam, New Zealand, Canada, and Australia (Rosser et al. 2008: 590). Most recently, scholars have identified the emergence of temporary spaces, called “pop-ups,” as a creative response to bar closures. Pop-ups are ephemeral, yet they provide enduring experiences of community and self-exploration (Bailey 2013; Moore 2016; Stillwagon and Ghaziani 2019).

Table 4.1 reviews the four streams of research in gayborhood studies, focusing on representative questions, major debates, and observational units. All adopt a macro, structural, or otherwise supra-individual lens of analysis and explanation.

**Table 4.1:** Research streams in gayborhood studies

Research stream	Questions	Debates	Observational units
Origins and ontology	Why do gayborhoods form? How do they change? Why do gayborhoods appeal to queer people? Why do they appeal to straight people?	Do queer people transform urban areas? Are economic or cultural forces more compelling explanations for the emergence and change of gayborhoods? Do places reflect forms of oppression?	Census tracts, community symbols, collective memories, real estate ads, business and non-profit listings, voting patterns
Organizational forms	What is the institutional profile of a gayborhood? What do they look like in different countries?	Do queer people comprise a “community” in a sociological sense? Do gayborhoods resemble ethnic enclaves? Do they have a global template?	Business, non-profit, and other organizational listings; overall institutional composition; pride parades, festivals, and other cultural events; cross-national comparisons
Technology	How do geo-coded mobile apps affect gayborhoods?	Do apps undermine queer spaces or creatively reconstitute them?	Mobile apps, online dating services, social media, HIV and STI infection rates
Change	Can a city have more than one gayborhood? Why are gay bars closing?	Are economic or cultural forces more compelling predictors of gayborhood change? Do gay bars still matter? Is spatial singularity or plurality a more valid description of urban sexualities?	Census tracts, real estate ads, business and non-profit listings, collective memories, revenues, nighttime economy, pop-ups, cultural archipelagos

Although scholars have produced considerable knowledge about gayborhoods, a key oversight remains: *what does the gayborhood mean for the people who actually live in it?* Neighborhoods are a “basic building block” of cities (Forsyth 2001: 343), but people relate to them and form attachments to them based on what they see and experience on the streets. By debating macro structural forces like gentrification, assimilation, technology, and demography, researchers who work in gayborhood studies elide matters of meaning, interactions, impressions, and interpretations. Whether a person finds the gayborhood significant—why it matters to them—is not a function of its statistical properties. A gayborhood is a collection of sentient people. To understand what it means, we need to ask people why they are drawn to it.

## Why Do You Live in the Gayborhood?

I draw on more than six hundred national media reports about the gayborhood across several decades of coverage, particularly stories in which a journalist interviewed local residents, to identify six major reasons why queer people say they live in a gay district and what about it appeals to them.^2^ Non-residential stakeholders make “vicarious” claims on gayborhoods as well (Greene 2014), but these are precisely what the concept of vicariousness suggests: proxy experiences that take the place of, or are imaged as related to, the ones of residents. The patterns of association, interactional styles, and perceptions among the people who actually live in a place, like the gayborhood, provide more valid access to its local knowledges (Geertz 1983) and meanings. I use the empirical expressions that residents offer to reflect theoretically on how urban sexualities acquire their significance on the streets—or what I call a “street empirics.”

Voting Blocs and Elections. Former San Francisco supervisor Harry Britt famously asserted that sexuality and space are inextricably linked: “When gays are spatially isolated, they are not gay, because they are invisible” (Castells 1983: 138). Gayborhood residents echo Britt’s intuition by focusing on the political effects of clustering. One San Franciscan said, “Having a specific neighborhood that politicians can point to, can go to and shake hands or kiss lesbian babies, has really solidified the gay vote, our political muscle.”^3^ By organizing themselves into an identifiable voting bloc, LGBTQ people can exert electoral influence. A story from the *New York Times* that covered the Congressional election of Nancy Pelosi noted, “The election Tuesday is being watched as a test of the cohesiveness and political strength of homosexuals.” Voter turnout showed that queer people helped to seat Pelosi, who had “campaigned frequently in homosexual neighborhoods.” Her campaign manager concluded, “It appears that homosexual voters contributed to her victory.” Pelosi received 20% of the vote in the Castro district.^4^

Former president Bill Clinton used a similar strategy. A story in the *New York Times* observed, “Voter-registration tables line gay neighborhoods. In discos, between videos of Madonna and the Pet Shop Boys, images flash on the screen of gay men and lesbians exhorting the crowds to vote. ‘Voting for Our Lives,’ say the signs in gay bars, bookstores and churches.”^5^ Another article in the same press reported on activity in San Francisco, where local officials “estimate that 95 percent of eligible voters are registered, in large part because of intensive voter-registration drives in gay neighborhoods.”^6^

The 2008 presidential race provides an example of the enduring capacity of gayborhoods to serve as voting blocs. A story in the *Windy City Times* reported, “Data available on voting in heavily gay precincts suggests the gay vote for Obama was at an unprecedented high. In the last several presidential elections, the percentage of LGB voters supporting the Democrat has hovered around 70 to 75 percent.” The ratio in the 2008 election was much higher. In Provincetown, 87% of the voters supported Obama, compared to 11% for [John] McCain. In San Francisco, 85% voted for Obama versus 13% for McCain. In Philadelphia’s gayborhood, 83% of voters supported Obama. He also won 89% of the vote in Dupont Circle, 63% of Dallas’s gay neighborhood, and 86% of Chicago’s Boystown.^7^

**Image 4.1:**
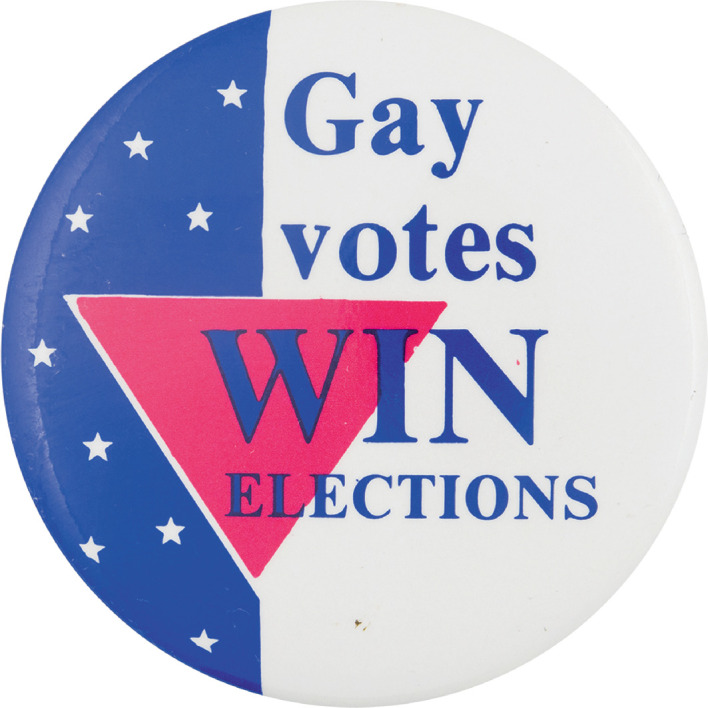
Voter registration drives in the gayborhood. Gay rights, gay votes campaign button (*Source* Image courtesy of: buttonmuseum.org)

**Image 4.2:**
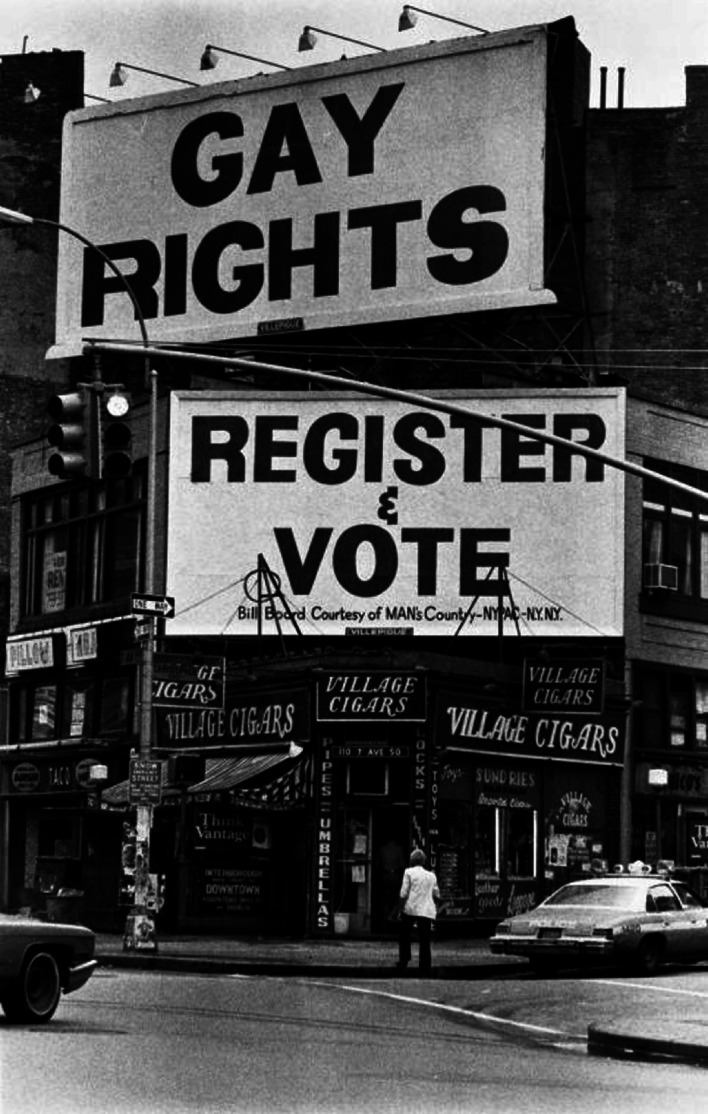
Gay rights and voter registration billboards at the corner of Christopher St. and Seventh Ave., Greenwich Village, New York City, 1978. The board was paid for by Man’s Country, a popular bathhouse chain that had branches in New York and Chicago in the 1970s and 1980s (*Source* Image © Bettye Lane. Photo provided by the New York Public Library. Reprinted with permission)

LGBTQ people are more interested in politics, more interested in public affairs, and more likely to be engaged in civic and political activities than their heterosexual counterparts (Egan et al. 2008). The examples that I have provided in this section suggest that the queer vote is often a determining factor in elections. During election cycles, gayborhood residents historically have often worn buttons on their bags to proclaim the power of their vote, and they have organized voter registration drives on the streets as well (Images 4.1 and 4.2).

Sex and Love. Because homosexuality is not universally or unambiguously visible on the body, queer people encounter unique challenges in finding each other for sex, dating, and mating. Gayborhoods can make things a little easier. The *New York Times* interviewed residents of Greenwich Village who reflected on what drew them to the neighborhood before it gentrified: “Older residents recall another era, when the street was paved not with gold, but with gays. That was what put Christopher Street on the cultural map, the old-timers say wistfully. ‘It was one big cruising street,’” said one resident who has lived in the neighborhood since the 1960s. The journalist added, “Gay men (the area never attracted a large lesbian population) carried the sidewalks as late as 1990, turning the street into a genuine carnival day and night. The waterfront, once a desolate truck yard, was a 24-hour playground of sexual trysts and flamboyant acts. By day, nude sunbathers staked out an urban beach on disfigured docks…‘ Straight people avoided Christopher Street,’” said the same resident, because it was “America’s gay Main Street.”^8^ Residents like these depend on the streets of gayborhoods, which are often shielded from the heterosexual gaze, to connect with each other.

**Image 4.3:**
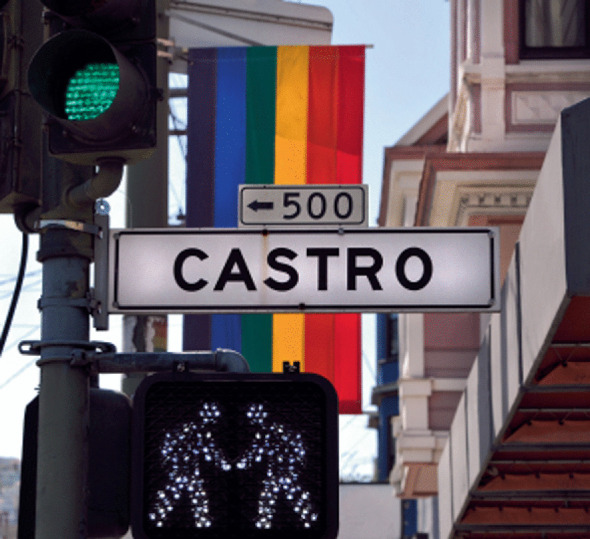
Hooking up—for a night or a lifetime (*Source* Graphic design by Graham Gremore for *Queerty*. Reprinted with permission)

Nearly four decades later and across the country, people still appreciate the streets of gayborhoods for their sexual networking opportunities. An editorial in the *Advocate* reflected on West Hollywood’s twentieth anniversary as “America’s first gay city” (it was incorporated on November 29, 1984): “I’m not arguing that West Hollywood is a perfect city, or even a gay mecca. But it is a special place…Whatever its flaws, it was a city that let people be themselves and make their own choices about whom they loved and how, without judgment or condemnation or shame.”^9^ A reporter from the *Village Voice* summarized a sensibility he heard from residents across the country: “Like any identity group, gay men and lesbians want to be with their own kind. It’s also easier to hook up—for a night or a lifetime.”^10^ Artistic renderings of this theme depict a same-sex couple in traffic lights in the gayborhood (Image 4.3).

Safe Spaces. Despite the statistical liberalization of attitudes toward homosexuality across the country (Twenge et al. 2015), many queer people find that the streets of gay neighborhoods feel safer than elsewhere in the city. Bob Witeck, CEO of Witeck Communications, Inc., a public relations and marketing communications firm that specializes in the queer consumer market, offered an observation based on his interactions with clients: “‘It’s about whether you can hold your partner’s hand in public, whether you’re safe from harassment or physical violence.’”^11^ Brian Orter, a photographer and commercial lighting designer who lives in Hell’s Kitchen, agreed. “I remember growing up in the city being gay in the ‘70s and ‘80s, and it was scary. So, I’m not going to go and move into a neighborhood where I am scared. I want to be near Chelsea and the West Village, where there are safe, gay people.”^12^ A reporter from the *Washington Post* compared the gayborhood with Ellis Island: “That’s what Greenwich Village has always been. A kind of Ellis Island for generations of gay men and lesbians…[W]hat it provided was freedom.”^13^ Although the gayborhood shifted from the Village to Chelsea, the sense that its streets were safer followed it, as this passage from the *New York Times* suggests: “Chelsea has become the gay neighborhood because gays and lesbians feel comfortable here.”^14^

The safe space theme resonates among younger generations as well. A reporter for the *Philadelphia Daily News* interviewed a high-school senior who “felt like she was home yesterday, walking the streets in the Gayborhood during OutFest, the Philly Pride event held each year on National Coming Out Day. But she’s not ‘at home’ in her house. Her ‘very Christian’ parents are unaware that she’s a lesbian, the 17-year-old said. ‘In my area, it’s very conservative—going to these places is very freeing because you can be yourself here. It feels like you’re not alone.’”^15^

Safety is a pronounced concern for queer youth of color. A writer for the *New York Times* notes, “For as long as Darnell could remember, the western edge of Christopher Street, with is rotting piers and dark alleys, had been a refuge for so-called pier kids like him. Black and Latino, and often from poor families that reject them for being gay, they are drawn to the street’s bleak fringes by a need to define themselves through the company of soul mates…‘Where I come from, you can’t be black and gay,’ said Darnell. ‘So we call this our home.’”^16^ Twenty-one-year-old college student Antonio Jones felt similarly. A journalist for the *Chicago Tribune* observed, “Young gay men from the city’s South and West sides come to Boystown to visit the Center on Halsted [the LGBTQ community center], whose youth programs make them feel safe, affirmed, and valued.” Jones told the reporter that “many of the youth come from communities that historically have been hostile to gays” who then “ find in Boystown a refuge. Often, it’s the first time the teens, the majority of whom are black, really can be themselves.”^17^

**Image 4.4:**
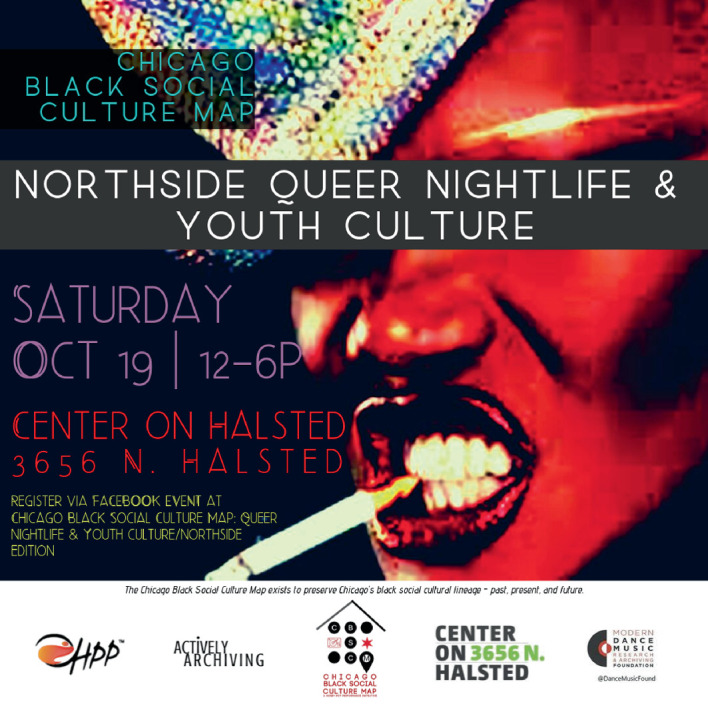
Queer youth culture (*Source* Graphic design by Kimeco Roberson and Chicago Black Social Culture Map. Reprinted with permission)

Image 4.4 shows an ad for a public programming event in Boystown that was produced by Honey Pot Performance, an Afro-diasporic feminist collaborative in Chicago that uses artistic expressions to examine questions of identity, belonging, community, and difference. Co-sponsored in 2019 by the Chicago Black Social Culture Map, the Modern Dance Music Archiving Foundation, and the Center on Halsted, the event included community archiving on site, oral histories, and panel discussions that celebrated nightlife’s queer roots, reflected on the significance of public events like Black Pride, and explored the importance of iconic spots and “anchor institutions” (Ghaziani 2014a: 383) in the gayborhood. The collaborators engaged with community members to collectively “tackle some of the challenging issues of black, brown and white queer communities all navigating nightlife together in the defined space of Boystown.”^18^

The media vignettes and current events that I have curated in this section remind us that the idea of safety underlying the popular notion of “ safe spaces” is relational, context dependent, and constructed through the collective experiences of people interacting with others on the streets.^19^

The Pink Economy. When gayborhoods were first forming, many people who moved there saw themselves as members of a minority group who needed to take care of each other—not just socially but also in an economic sense. Bars, bathhouses, bookstores, and other businesses that targeted a queer niche market emerged to service the newly visible residents (Ghaziani 2015b). A journalist writing for the *Advocate* interviewed Elmwood Hopkins, managing director of Emerging Markets Inc., a consulting firm in Los Angeles. Hopkins remarked on the historical arc of the pink economy, offering important lessons for urban planners who try to either preserve or reinvigorate neighborhoods:

Most urban planners try to revive neighborhoods in a backward manner by building affordable housing and then hoping people move into the area. Instead, he says, restaurants, shops, art studios, and other services should be there first. Then the residents will come. Gay men and lesbians realized that years ago, he says, when in the 1920s and 1930s they gravitated toward certain neighborhoods in cities across the United States. Their presence led gay bars and other businesses to open, and then more residents arrived.^20^

The pink economy gained momentum as gayborhoods became more institutionally complete. “We’re at a tipping point, with gays coming out in society and business,” said a queer hospitality consultant in San Francisco to the *USA Today*. “All of a sudden, we’ve become a great market for all industries to go after.”^21^ Peak visibility arrived on June 2, 2004 when the Greater Philadelphia Tourism Marketing Company (GPTMC) launched a multimillion-dollar television campaign to lure lesbian and gay tourists to their city. On a winter afternoon in 2003, in a conference room that overlooked the Ben Franklin Bridge, six marketing strategists met and devised a catchy campaign: “Get your history straight and your nightlife gay.” The ad made Philadelphia “the first destination in the world to produce a gay-themed television commercial. Never before has a U.S. city, resort, or international destination used television advertising to invite gay travelers to visit.”^22^ The *Washington Post* described the ad:

‘My dearest beloved,’ the voice-over starts, as a presumably 18th-century fellow writes impassionedly in the television commercial. ‘How I long to be with you, to see your radiant smile. Please journey to Philadelphia, where we will be at liberty to meet this Monday, at Independence Hall, as the clock strikes 6.’ In the next scene, the man in period attire waits with flowers. An attractive girl flirts with him as she walks by. Then, another man sneaks up behind him and they walk away together. ‘Come to Philadelphia,’ the voice-over then says. ‘Get your history straight. And your nightlife gay.’^23^

**Image 4.5:**
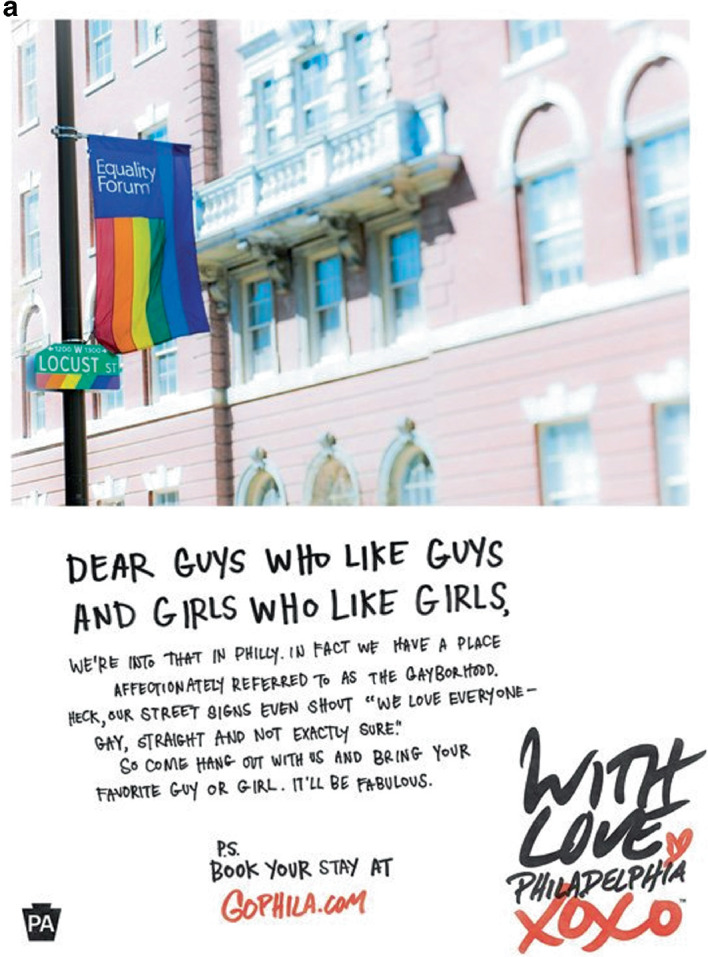

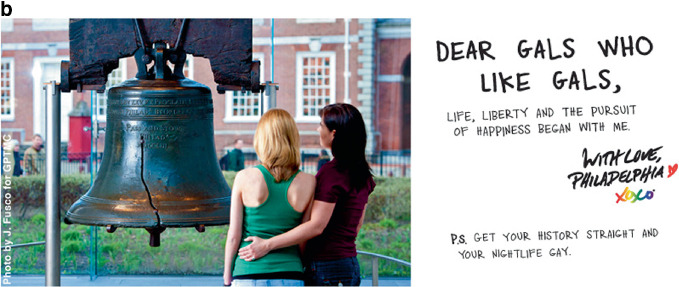

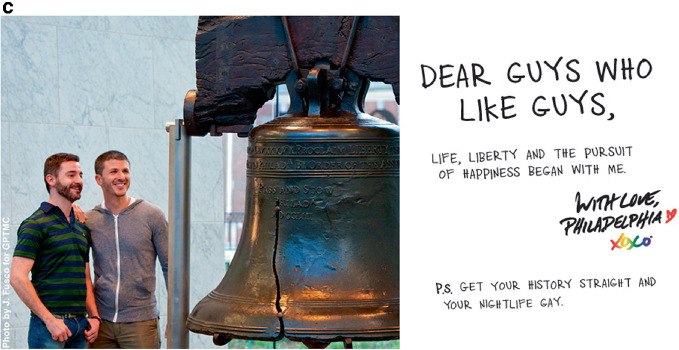
Get your history straight, and your nightlife gay (*Source* Images 4.5b and 4.5c by J. Fusco. All images reprinted with permission from Visit Philadelphia)

The success of the commercial motivated the city to produce a companion magazine ad as well. A front-page *Philadelphia Inquirer* article described the effort: “The theme is ‘Get your history straight and your nightlife gay.’” The three-year, $900,000 effort sought “to integrate Philadelphia’s historical and cultural offerings with gay-specific attractions.”^24^ The strategy worked; Philadelphia saw a $153 return for every dollar that it spent on its marketing campaign. Bruce Yelk, the Director of Public Relations, said that the ad took the “City of Brotherly Love” from an unranked position on Community Marketing’s “Top 10 U.S. Destinations for the LGBT Traveler” list to the number ten spot. Image 4.5 shows several expressions from the campaign. Philadelphia’s success motivated more than 75 cities around the world to adopt queer tourism campaigns.^25^

Activism and Protest. An incitement to insurgency requires people to define their situation as unjust and to feel optimistic about their prospects for change. This type of culture work—redefining what a situation means—happens on the ground in specific places. Consider an example from Dade County (Miami). Former beauty queen (Miss Oklahoma, 1958, and second runner-up for Miss America, 1959) and recording artist-turned-born-again Christian evangelist mother Anita Bryant became involved in a campaign called “Save Our Children.” Bryant proclaimed that “the recruitment of our children is absolutely necessary for the survival and growth of homosexuality.” She personalized her message by expressing concern over the wellbeing of her own children: “As a mother, I know that homosexuals cannot biologically reproduce children; therefore, they must recruit our children.” As part of her campaign and with the aid of fundamentalist churches and conservative Roman Catholic groups, Bryant displayed images from San Francisco’s pride parades and argued that the city was “a cesspool of sexual perversion gone rampant.” Shen then cautioned local voters, “ Don’t let Miami become another San Francisco.” Residents found her message compelling and voted by a margin of more than 2-to-1 in a referendum to repeal a law that protected gay men and lesbians from discrimination in employment, housing, and public accommodation (Ghaziani 2008: 33).

The Florida fight unleashed protests across the country, many of which were organized in gay neighborhoods. A *Washington Post* story observed, “A gay cause can quickly become a neighborhood cause. Soon after Anita Bryant’s recent victory in a Miami homosexual rights referendum, most of the restaurants around Dupont Circle agreed—some without prodding—to stop serving the Florida orange juice Bryant advertises.”^26^ The protest theme found its way to the first national March on Washington for Lesbian and Gay Rights in 1978.

In another well-known example, the San Francisco queer community united when Dan White assassinated supervisor Harvey Milk and Mayor George Mascone on November 27, 1978. A front-page story in the *New York Times* described the power of gayborhood streets for social movement mobilization efforts: “While the Castro has been the center of a movement, it is also home to ‘an important political constituency. When people were angry about Dan White, they were able to assemble quickly, spilling out of bars [into the streets]…Physical location mattered.’”^27^ White received a lenient sentence of voluntary manslaughter. Outrage in San Francisco’s gay and lesbian community sparked the “White Night riots” on May 21, 1979. Protesters set ablaze eleven police cars and smashed the windows of City Hall, holding up placards that read, “Did Harvey Milk Die for Nothing?”

In the 1980s, queer communities across the United States used gayborhoods to respond to the AIDS crisis. A reporter for the *New York Times* commented, “Sociologists and demographers alike say the concentration of homosexuals in core neighborhoods has grown in the last two decades out of gay political advocacy and the AIDS crisis.”^28^ A writer for the *San Francisco Chronicle* added that mobilization in gay districts helped to lower infection rates: “When AIDS finally was identified, white middle-class gays mobilized powerfully, and over time their efforts drove down infection rates in San Francisco’s Castro district.”^29^

In response to escalating anti-gay hate crimes in the 1990s, queer people again used their residential concentration in gayborhoods to redefine their situation as unjust and to respond to it. Spikes in gay bashing and murders “accelerated our plans to do something to take back our streets,” one New Yorker said. Another remarked, “It’s one horror story after another. Every day I hear about a friend or someone I know getting hurt. My lover and I were almost physically attacked in the East Village. We’re verbally harassed all the time, called ‘dykes’ and ‘ queers’ and ‘what’s wrong with you’…Our message is, ‘we’re bashing back.’”^30^

 Christopher Street residents formed a group called the “Pink Panthers,” a neighborhood foot patrol who monitored city streets. Writing for the *Washington Post*, Paula Span remarked on group’s name, logo, and activities:

They could have called themselves something more prosaic, neighborhood anti-crime patrols being nothing new, after all…But gay activism, New York-style, requires a certain ironic panache…The Pink Panthers title, with its echoes both of ‘60s politicization and silver-screen camp, won swift approval. The group’s logo – an inverted pink triangle bearing a paw print – was invented that very night. In the few weeks since, says founder Gerri Wells, about 150 people have volunteered to join the Panthers’ weekend patrols. From midnight until 3 a.m. on Friday and Saturday nights, armed only with whistles and squawky CB radios and a series of training sessions, patrols of eight to 10 people in paw-printed black T-shirts stride through the West Village. They watch; they jot down license plate numbers; they call the police if they see trouble; they blow whistles to scare off assailants; they intervene to extricate victims.^31^

The Pink Panthers provide “a searing response to the increased violence that has accompanied the general increase of gay visibility in America” (Berlant and Freeman 1993: 206). Activists appropriated confrontational strategies of the black power movement—but with a twist: “Dressed in black T-shirts with pink triangles enclosing a black paw print, they move unarmed in groups, linked by walkie-talkies and whistles. In choosing a uniform that explicitly marks them as targets, [they identify themselves] as successors of the Black Power movement” (ibid.). The Panthers cultivated their consciousness, and executed their protest campaigns, on gayborhood streets (Image 4.5).

**Image 4.6:**
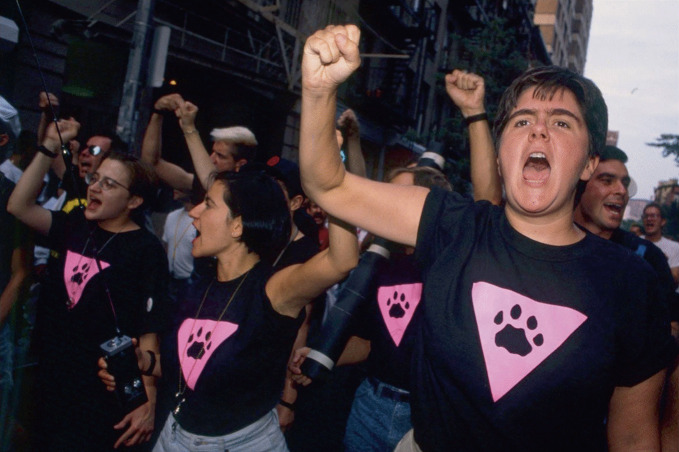
The Pink Panthers (*Source* T.L. Litt. Reprinted with permission)

 Gayborhoods became a base camp for marriage protests as well. The LGBTQ movement put marriage on its national agenda for the first time in 1987 at its third March on Washington. Couples, Inc., a Los Angeles-based organization fighting for legal recognition of gay partners, organized The Wedding, a ceremony that celebrated queer relationships and demanded that their partnerships receive equal legal recognition as married heterosexuals (Ghaziani 2008). Three years after the Washington march, two lesbian couples and one gay male couple in Hawaii applied for marriage licenses. Like others, they were refused. Unlike others, however, they filed a law suit against the state for denying their civil rights. In 1993, the Supreme Court of Hawaii decided *Baehr v.* *Lewin*—a “ruling that roiled America” (Sullivan 1997: 104)—and declared that the denial of marriage licenses on the grounds of same-sex applications violated the equal protection clause of the state’s constitution that outlawed sex-based discrimination.

Fearing the effects of the ruling, California republican William J. Knight introduced a bill that would invalidate “any marriage contracted outside this state between individuals of the same gender.” The bill passed the Assembly 41 to 33 on January 31, 1996. In response, a protest group called the Freedom to Marry Task Force of Northern California “collected 1,600 letters in the heart of San Francisco’s largest gay neighborhood, opposing Mr. Knight’s bill.” One member commented on why the gayborhood mattered for their actions: “When we stand there [in the Castro] with the Freedom to Marry banner, people swarm over.”^32^ These early campaigns motivated activists to jump into the fray and organize for marriage equality (see Ghaziani et al. 2016 for review).

From Anita Bryant to Dan White, and from the AIDS crisis to hate crimes and marriage equality—each of these examples shows with particular force how spatial concentration cultivates political consciousness and protest. All of this happens on the streets. Building gayborhoods was “inseparable from the development of the gay community as a social movement” (Castells 1983: 157). In today’s climate of greater legislative equality, gayborhoods provide an abeyance (Taylor 1989) functionality, allowing queer people to stand on guard and ready to resist any injustices that may come their way.

Community Building. Like attracts like. This is a well-established fact of human life, one that sociologists call homophily. Geography is a key precondition for homophily. “We are more likely to have contact with those who are closer to us in geographic location than those who are distant” (McPherson et al. 2001: 429). These academic insights filter down to the streets of the gayborhood as well. One resident from Asbury Park, NJ explained why she moved to the area: “There’s an acceptance here, a feeling of community, and there are a few gathering places for gay and lesbian people.”^33^ A New Yorker similarly pointed to the social aspects of seeking community in the city: “This is the only place to be ourselves, to be with people who are like ourselves and not be looked down on.”^34^ A journalist for the *Village Voice* offered the same observation: “Like any identity group, gay men and lesbians want to be with their own kind.”^35^

Whereas the social aspects of community building point to the relational benefits that gayborhoods provide, the cultural component highlights the symbolic and expressive aspects of its streets. Regina Quattrochi, the former director of the New York City AIDS Resource Center, argues that gayborhoods have always promoted the celebration of queer cultures: “Even as recently as the early and mid-1980s, I think the Village was symbolic of a sort of celebration of gay culture.”^36^ The *Washington Post* playfully compared gayborhoods to Oz:

For decades, the gay neighborhoods of San Francisco, New York, and Washington embodied the promise of change, freedom, friendship, and acceptance. Greeting cards and T-shirts were emblazoned with the slogan ‘I have a feeling we’re not in Kansas anymore.’ To come out of the closet, to move to those gay utopias, was to be swept up by a tornado and dropped into Oz. The black-and-white landscape dissolved into color…Reborn, gay men often find that old assumptions about family, love, and community fall away as well. In the ‘70s, men once derided as sissies remade themselves into ‘Castro clones,’ with cowboy boots and button-fly Levi’s, plaid shirts and leather jackets, and studiously well-muscled bodies.^37^

**Image 4.7:**
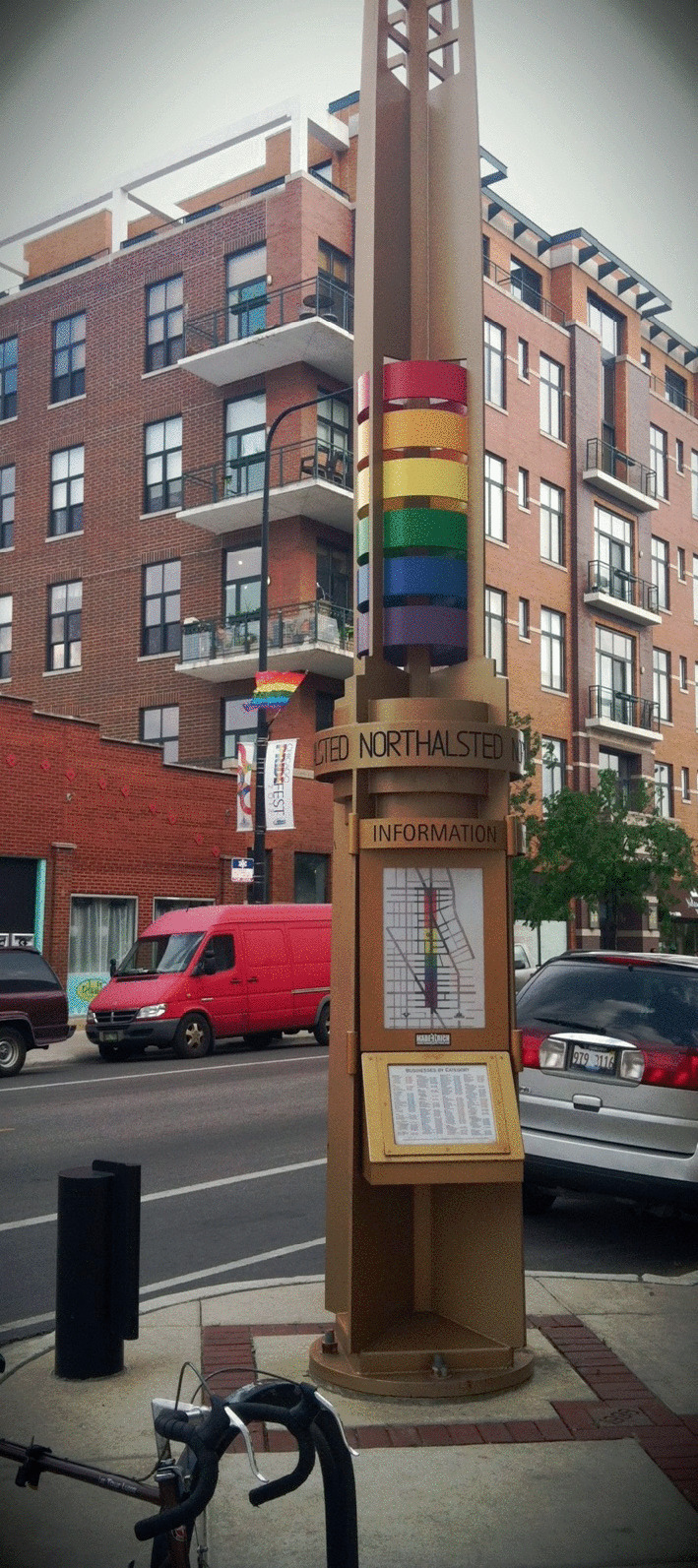
Rainbow pylons in Chicago’s gayborhood (*Source* Photo by Gary Baker. Reprinted with permission)

We also hear the importance of community building in debates about whether to municipally mark gayborhoods. In 1997, Chicago became the first city in the world to use tax dollars to formally designate a section of its East Lakeview neighborhood as “Boystown.” It did so by installing rainbow-colored art deco pylons along North Halsted Street (Image 4.7).

A local paper published a front-page editorial article that expressed skepticism about the city’s decision: “Why should a neighborhood have a public sexual designation when sex is the ultimate private act? Why would gay people want to officially ghettoize themselves when they’ve fought so hard not to be ostracized?” The writer interviewed Tracy Baim, who managed local queer periodicals, for answers. In her response, Baim combined several themes from this chapter:

The city’s plan isn’t about sex, it’s about community. Society has forced us to define ourselves as a community to protect ourselves…Community has given gays the force to fight against hate crimes, against job discrimination and housing bias. The gay community has become family for gays whose families have thrown them out. The city’s plan simply would recognize that community, along with the work it has done to turn the neighborhood into a place where straight people, along with gays, want to shop, eat and live. Why does the city do it for Chinatown? Why does it do it for Greektown? Because it helps bring pride to an area of town that has traditionally been built by those communities.^38^

Richard Daley, who was mayor at the time, agreed: “I knew from the beginning it was about fairness—fairness to this community. I am thanking you for what you (the LGBTQ community) have done for North Halsted Street for many, many years.”^39^

**Image 4.8:**
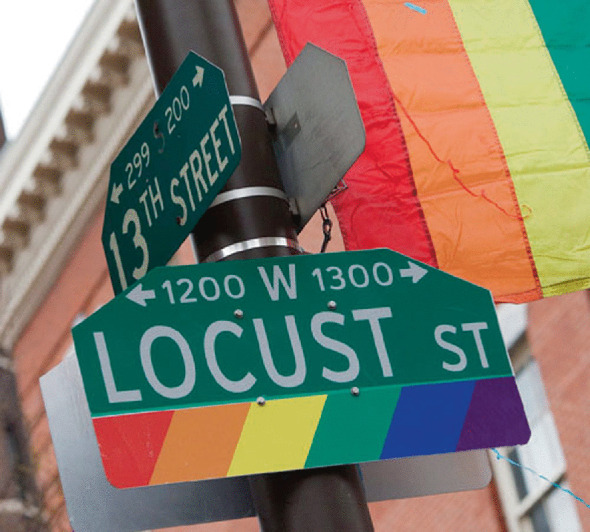
Rainbow street signs in Philadelphia (*Source* Photo by J. Smith. Reprinted with permission from Visit Philadelphia)

A similar conversation happened in Philadelphia ten years later when Mayor John F. Street dedicated thirty-six new street signs to celebrate the city’s queer community (Image 4.8).

The *Philadelphia Daily News* remarked on the significance of the street signs:

‘Welcome to the ‘Gayborhood.’ A welcoming vibe is what organizers hope to inspire when visitors see new street signage that will designate a portion of the Center City District as the city’s official gay, lesbian, bisexual and transgender-sensitive neighborhood…the new street signs will feature the traditional GLBT rainbow, or ‘Freedom’ flag underneath the usual street signs…‘The signage is an important symbol for this city,’ [said Tami Sortman of the Philadelphia Gay Tourism caucus]. ‘We can say that we have a neighborhood…Not only does this bring a sense of welcoming to the local community, it’s a symbol to the world.’^40^

The image of gayborhoods as a cultural mecca occurs repeatedly in the national media. A front-page story in the *San Francisco Chronicle* from 1996 quoted a Castro resident who said, “I knew I had to get out of Nebraska in 1971. San Francisco was a mecca for gay people like me.” A year later, the same press described the Castro as a place that “drew thousands of gays from all over the country because they believed it was their own mecca-in-the-making.” By 1999, it declared that the district was “the world’s gay mecca.”^41^

 San Francisco is not alone in its use of the imagery of mecca. Some reporters describe Provincetown, MA as “a gay mecca in the summer months,” while New Yorkers add, “To the old-timers, Christopher Street was, and should stay, New York’s Gay Mecca, where the promise of liberation remains alive.”^42^

In 1994, New York commemorated the 25th anniversary of the Stonewall riots. That year, the *Washington Post* ran a poignant story, worth quoting at length, that blended Islamic and American images to celebrate its gayborhoods:

There will be a constant stream of pilgrims coming to gaze at the brick-and-stucco facade of the Stonewall over the next few days. Because a police raid turned into a riot there 25 years ago, because the patrons of a gay bar did not go gently into a paddy wagon, hundreds of thousands of people will descend on New York for a weekend of commemoration. The neighborhood surrounding the old saloon, a hangout-turned-landmark, will become an international mecca, a symbol of gay liberation.

But that’s what Greenwich Village has always been. A kind of Ellis Island for generations of gay men and lesbians, a crucible of gay history since before the Jazz Age, it is America’s most celebrated gay enclave. What the Village offered was a handful of places where gay people could reveal themselves: a cafeteria here, a bar there, a park, a bookstore, eventually a community center. But what it provided was freedom. ‘It’s a mythic place,’ says Joan Nestle, co-founder of the Lesbian Herstory Archives.

Sometime in the 1970s, San Francisco’s Castro district eclipsed the Village as a national mecca and a political power base… Other New York neighborhoods have drained away some of its functions. The gay middle class has largely decamped for Chelsea, a few blocks uptown, which now boasts blocks of new restaurants, bars and boutiques. The most vibrant lesbian community in the city is across the river in Brooklyn’s Park Slope. And the crowd that generates performance art, cutting-edge music, fashion and attitude is headquartered in the East Village. Yet the neighborhood’s hold on the imagination remains powerful. And this weekend, it will again be at the heart of everything.^43^

The use of religious imagery to characterize gayborhoods is ironic but unsurprising. At the heart of any spiritual iconography is a communal affirmation (Durkheim 1912). An editorial from Chicago echoed, “Our eroticism is the closest thing we have to what in the past was called a spiritual life, and no one wants to be excommunicated from that church altogether. This is probably why people who are seen or see themselves as primarily homosexual have acceded to their own subculturalization in gay ghettos.”^44^ In this sense, gayborhoods resemble the totems that Durkheim described in his study of religious life. In both instances, there is a common motivation to seek the sacred and celebrate as its source ourselves and our communities. This type of work—from socializing to community building and transcendence—happens on the streets of gayborhoods as people interact with their neighbors, visitors, and tourists alike.

## Conclusions

In this chapter, I identified six major reasons that queer people have shared with journalists across the United States about why gayborhoods matter to them. My findings show that gay districts provide access to courtship and partnership possibilities, influence elections, provide a perception of safer streets, offer access to queer businesses and institutions, enable social movement organizing, and are the conduits of community building. Together, these residential logics provide insights into the motivations, meanings, interpretations, and interactions that uniquely happen on the streets of gay neighborhoods (Table 4.2).

**Table 4.2:** The street empirics of the gayborhood

Residential logics: Why do you live in the gayborhood?	Street empirics: Why do gayborhoods matter?
Voting blocs and elections	“Having a specific neighborhood that politicians can point to has really solidified the gay vote, our political muscle”
Sex and love	“Gay men and lesbians want to be with their own kind. It’s easier to hook up—for a night or a lifetime”
Safe spaces	“It’s about whether you can hold your partner’s hand in public, whether you’re safe from harassment or physical violence”
The pink economy	“Restaurants, shops, art studios, and other services should be there first. Then the residents will come”
Activism and protest	“When AIDS finally was identified, white middle-class gays mobilized powerfully, and over time their efforts drove down infection rates in San Francisco’s Castro district”
Community building	“That’s what Greenwich Village has always been. A kind of Ellis Island for generations of gay men and lesbians”

Research in gayborhood studies often assumes that we need to isolate macro structural factors like the economy (e.g., real estate values), culture (e.g., assimilation), politics (e.g., legislation and public opinion), and technology (e.g., geo-aware apps like Grindr) to study these urban districts. This assumption originates from dominant theoretical traditions in urban sociology (e.g., Abrahamson 2005; Castells 1976; Logan and Molotch 1987; Molotch 1976; Orum and Chen 2003; Sassen 2001; Zukin 1987), especially the Chicago School (Park 1915; Park and Burgess 1925).

A “supra-individual” approach (Sampson 2012: 23) like this, and the assumptions that it forces researchers to make, persists in contemporary research about the city as well. Consider a recent call by Wu (2016: 126) that “ urban sociology should be understood as the sociology of city.” By making this move, scholars would focus less on “social problems within an urban context”—like influencing elections, finding a sexual or romantic partner, feeling safe, looking for specialty stores or non-profits, mobilizing against real or perceived threats, and desiring the company of similar others—and instead analyze “the city as an autonomous social unit” (ibid.). Wu’s recommendation is provocative, and productive, but unless we texture our impressions of the city with the meaning-making processes that happen on the ground—a street empirics, as call it—our knowledge will be incomplete. For Wu, the goal is “treating the city as the unit of analysis” (ibid.), but this mandate will also abstract our view too far away from the streets.

In this chapter, I have called on Urbanists to embrace an analytic strategy of street empirics. Those sidewalks where people walk, talk, and interact with each other provide a foundational unit of analysis for scholars who are interested in understanding what a place means to its residents. By accepting this methodological directive, we can use the reasons that gayborhood residents provide for why they live in the area, like other residents in other neighborhoods, to explain the significance of a place.

My call for prioritizing street empirics to understand what a neighborhood means—why it matters to the people who live there—enables scholars to think broadly about the interactional and attitudinal mechanisms that produce place characters. As an analytic approach, street empirics is methodologically robust. Consider that I write these words in the middle of a pandemic. Covid-19 has motivated many people to recalibrate how and why places influence them. One headline wondered about the significance of cities: “ Coronavirus may prompt migration out of American cities.” Others mused about queer cultures—“Of Pride in Pandemic Times”—and queer spaces: “Can LGBTQ bars survive the Covid-19 pandemic?”^45^ My data predates the pandemic, but I imagine that future researchers can still use streets empirics to systematically analyze how Covid-19 affected the meanings of urban gay districts. Moments of crisis compel creative responses, and we now have another approach in our toolkit that we can use to advance gayborhood studies.

## Footnotes

1 There is a separate body of work on rural and suburban sexualities (e.g., Bell and Valentine 1995; Brekhus 2003; Fellows 1996; Forsyth 1997; Gray 2009; Kazyak 2012). We can debate whether to subsume these ideas under gayborhood studies. Although they sometimes have unique theoretical debates, many scholars also offer an anti-urban challenge to queer metronormativity (Halberstam 2005; Herring 2010). Gayborhood studies would be incomplete without these critiques.

2 For more information about this data set, see (Ghaziani 2014b). The public conversation that it represents includes 27 urban, suburban, and rural locations, and it spans 40 years of coverage (1970 to 2010). For a companion discussion about why straight people say they want to live in a gayborhood, see (Ghaziani 2019d).

3 “S.F.’s Castro District Faces an Identity Crisis,” by Wyatt Buchanan. *San Francisco Chronicle*, February 25, 2007, page A1.

4 Pelosi quote: “Homosexuals’ Political Power Tested in the West,” by Robert Lindsey. *New York Times*, April 5, 1987, section 1, page 1. Pelosi’s vote in the Castro: “House Race in West Goes on to Runoff,” by Robert Lindsey. *New York Times*, April 9, 1987, page A14.

5 “Gay Politics Goes Mainstream,” by Jeffrey Schmalz. *New York Times*, October 11, 1992, section 6, page 18.

6 “ Voting Scared,” by Jeffrey Schmalz. *New York Times*, November 1, 1992, section 4, page 1.

7 “Obama’s Win and the Gay Vote,” by Lisa Keen. *Windy City Times*, November 5, 2008.

8 “Street Fight,” by Denny Lee. *New York Times*, March 31, 2002, section 14, page 1.

9 “WeHo, Warts and All,” by John Morgan Wilson. *Advocate*, December 21, 2004, issue 929, page unnoted.

10 “The New Gayborhoods of Fort Greene, Sunset Park, and Jackson Heights,” by Michael Lavers. *Village Voice*, June 24, 2009, page unnoted.

11 “Cities Seek Lucrative Gay Tourist Dollar,” by Macenzie Carpenter. *East Bay Times*, February 2, 2007.

12 “Under the Rainbow,” by David Shaftel. *New York Times*, March 25, 2007.

13 “Greenwich Time: On the Stonewall anniversary, a gay tour of Village history,” by Paula Span. *Washington Post*, June 22, 1994, page D1.

14 “Gay Businesses Follow Influx of Gay People,” by Marvine Howe. *New York Times*, April 10, 1994, section 14, page 8.

15 “For Some, Coming Out is Like Finding Home,” by Regina Medina. *Philadelphia Daily News*, October 12, 2009, page 6.

16 “Street Fight,” by Denny Lee. *New York Times*, March 31, 2002, section 14, page 1.

17 “Boystown Grapples with Black Youth Influx,” by Dawn Turner Trice. *Chicago Tribune*, November 30, 2009, page 6.

18 https://www.honeypotperformance.org/events/2019/10/19/chicago-black-social-culture-map-queer-nightlife-amp-youth-culture-northside-edition.

19 For a challenge to the argument that gayborhood streets feel safe for queer people of color, see (Hanhardt 2013).

20 “There Goes the Gayborhood,” by Fred Kuhr. *The Advocate*, July 6, 2004, pp. 34–36 (quote on page 35).

21 “More Marketing Aimed at Gay Consumers: Companies covet their buying power,” by Edward Iwata. *USA Today*, November 2, 2006, page B3.

22 “Ground-breaking TV Ad Set to Promote ‘ Gay-Friendly’ Philly,” by John Fischer.

23 “Philadelphia Invites Gay Tourists in TV Ad,” by Robert Strauss. *Washington Post*, July 3, 2004, page A03.

24 “New Tourism Ads: Come out and visit,” by Marcia Gelbart. *Philadelphia Inquirer*, November 14, 2003, page A01.

25 “Philadelphia Strikes Gold Catering to Gay Tourists,” by Rubina Madan. *Chicago Sun Times*, August 5, 2007, page C2. Yelk quote: https://www.phillymag.com/news/2013/11/13/get-history-straight-nightlife-gay-campaign-turns-10-today/.

26 “The Gay Life at Dupont Circle,” by Robert F. Levey. *Washington Post*, June 16, 1977, page DC1. See also coverage in the *New York Times*: https://www.nytimes.com/1977/06/08/archives/miami-votes-2-to-1-to-repeal-law-barring-bias-against-homosexuals.html.

27 “ Gay Enclaves Face Prospect of Being Passé,” by Patricia Leigh Brown. *New York Times*, October 30, 2007, page A1.

28 “Gay Presence Leads to Revival of Declining Neighborhoods,” by Karen De Witt. *New York Times*, September 6, 1994, page A14.

29 “AIDS Fear has Faded in U.S.,” by Marshall Kilduff. *San Francisco Chronicle*, July 16, 2004, page B8.

30 Take back our streets: “Streets of Sanctuary Now Harbor Criminals,” authorship unnoted. *New York Times*, August 6, 1990, page B1. Bashing back: “Patrol of the Pink Panthers,” by Paula Span. *Washington Post*, September 19, 1990, page C1.

31 “Patrol of the Pink Panthers,” by Paula Span. *Washington Post*, September 19, 1990, page C1.

32 “Fearing a Toehold for Gay Marriages, Conservatives Rush to Bar the Door,” by David W. Dunlap. *New York Times*, March 6, 1996, page A13.

33 “Move Over, Fire Island, Here Comes Asbury Park,” by Jill P. Capuzzo. *New York Times*, August 6, 2000, section 14NJ, page 1.

34 “Race, Class, and Sex Breed Contempt in Greenwich Village,” by Michelle Garcia.

35 “The New Gayborhoods of Fort Greene, Sunset Park, and Jackson Heights,” by Michael Lavers. *Village Voice*, June 24, 2009, page unnoted.

36 “Neighborhood Report: West Village,” by Randy Kennedy. *The* *New York Times*, June 19, 1994, page 6.

37 “Fill-in-the-Blanks: For gay men, self-invention can be a key to self-discovery,” by Elizabeth Kastor. *Washington Post*, July 31, 1997, page B01.

38 “Gaytown Enters Gray Area of Community Naming,” by Michael Schmich. *Chicago Tribune*, August 17, 1997, page 1.

39 “Gay-Pride Halsted Street Project Ends in Harmony,” by Lola Smallwood. *Chicago Tribune*, November 15, 1998, page 3.

40 “New Signs Make it Official: We have a ‘Gayborhood,’” by Damon C. Williams. *Philadelphia Daily News*, April 19, 2007, page 22.

41 1996: “There Goes the Neighborhood: After 25 years at the heart of San Francisco’s gay movement, the Castro district is going mainstream,” by Dan Levy. *San Francisco Chronicle*, May 26, 1996, page 1/Z1. 1997: “A ‘Soft-Focus’ Look at the Castro,” by Dan Levy. *San Francisco Chronicle*, March 15, 1997, page E1. 1999: “Market Street’s Proud History is at Stake,” by Ken Garcia. *San Francisco Chronicle*, May 20, 1999, page A17.

42 “A Vacation Gone to the ‘Dogs,’” by Mick LaSalle. *San Francisco Chronicle*, July 14, 1995, page C4. “Street Fight,” by Denny Lee. *New York Times*, March 31, 2002, section 14, page 1.

43 “Greenwich Time: On the Stonewall anniversary, a gay tour of Village history,” by Paula Span. *The Washington Post*, June 22, 1994, page D1.

44 “Bringing Clarity to an Elusive Reality,” by Joseph Coates. *Chicago Tribune*, May 2, 1991, page 3.

45 Migration out of cities: https://theharrispoll.com/coronavirus-may-prompt-migration-out-of-american-cities/; Pride: https://shepherdexpress.com/lgbtq/my-lgbtq-pov/of-pride-in-pandemic-times/; LGBTQ bars: https://www.bizjournals.com/sanfrancisco/news/2020/06/12/can-lgbtq-bars-survive-through-covid-19.html.
